# Prognostic assessment for patients with cancer and incidental pulmonary embolism

**DOI:** 10.1186/s12959-017-0157-x

**Published:** 2018-02-06

**Authors:** George Bozas, Natalie Jeffery, Deiva Ramanujam-Venkatachala, Ged Avery, Andrew Stephens, Hilary Moss, June Palmer, Mandi Elliott, Anthony Maraveyas

**Affiliations:** 1 Hull and East Yorkshire NHS Hospitals Trust, Queen’s Centre for Oncology and Haematology, Castle Hill Hospital, Castle Road, Cottingham, HU16 5JQ UK; 2 Hull and East Yorkshire NHS Hospitals Trust, Radiology Department, Castle Hill Hospital, Castle Road, Cottingham, HU16 5JQ UK; 3 Hull York Medical School, Academic Oncology, Castle Hill Hospital, Castle Road, Cottingham, HU16 5JQ UK

**Keywords:** Pulmonary embolism, Cancer, Incidental finding, Unsuspected pulmonary embolism, Prognosis

## Abstract

**Background:**

An incidental/unsuspected diagnosis of pulmonary embolism (IPE) in cancer patients is a frequent occurrence. This single-institution analysis of uniformly managed patients investigates short and long-term outcomes and proposes a prognostic risk score, aiming to assist clinical decision-making.

**Methods:**

Data from a prospectively recorded cohort of 234 consecutive cancer patients with IPE were analysed. Multivariate logistic regression and the Cox regression survival methods were used to identify factors with independent association with early (30-day, 3-month, 6-month) mortality and survival. Receiver operator characteristic analysis (ROC) was used to assess appropriate cut-offs for continuous variables and the fitness of prognostic scoring.

**Results:**

30-day, 3-month and 6-month mortality was 3.4% (*n* = 8), 15% (*n* = 35) and 31% (*n* = 72) respectively. Recurrence during anticoagulation occurred in 2.6% (*n* = 6) and major haemorrhage in 2.1% (*n* = 5) of the patients. A prognostic score incorporating performance status (0 vs 1–2 vs 3–4) and the presence of new or worsening symptoms, with and without the consideration of the presence of incurable malignancy, correlated with overall survival (*p* < .001 respectively) as well as early mortality (AUC = .821, *p* = .004 and AUC = .805, *p* = 0.006, respectively).

**Conclusion:**

A simple prognostic score incorporating basic oncologic clinical assessment and self-reported symptomatology could reliably stratify the mortality risk of ambulant cancer patients and IPE.

**Trial registration:**

Audit registration No. 2013.287, Hull and East Yorkshire Hospitals Trust, 29/11/2013.

**Electronic supplementary material:**

The online version of this article (10.1186/s12959-017-0157-x) contains supplementary material, which is available to authorized users.

## Background

The widespread use of multi-slice CT in diagnosis, staging and assessment of response to treatment has resulted in an increase in the apparent incidence of what has been termed unsuspected or incidental pulmonary embolism (IPE) in cancer patients [1–3]. Notably, up to half of vascular thromboembolic events (VTE) diagnosed in Oncology centres may be incidental [1, 4]. Contrary to the assumption that these cases are asymptomatic, contemporary work shows that for a substantial majority this perception is erroneous [4, 5]. Clinicians frequently attribute relevant symptomatology to the progression of underlying cancer or to the adverse effect of cancer treatments and often it will remain unclear indeed whether symptomatology might be attributable to the imaged PE, especially when progressive cancer is imaged concurrently. This remains a particularly unclear area as most of the data available are retrospective without nuance. Recent work seems to support the notion that cancer patients with IPE have similar outcomes to symptomatic (suspected) PE cases, their survival appearing worse compared with matched controls without PE [6–11]. Moreover, the presence of symptoms in cancer patients with IPE has been correlated with poorer outcome [12]. On the other hand it has been shown that some patients with IPE do not develop symptoms or morbidity [13].

The standard of care remains to treat all cancer patients with a PE or DVT irrespective of the manner of diagnosis [14, 15]. Outpatient care is commonly used but there is little evidence to underpin outpatient approaches, these often being empirical and based on individual clinician expertise. Care standards thus are often fragmented, poorly adhered to or vary between different specialities [16, 17]. In general it is recognised that management recommendations for IPE are extrapolated from studies on symptomatic VTE [18] and evidence from retrospective studies. Additional controversy exists for distal IPE (affecting segmental or subsegmental pulmonary artery branches); a recent meta-analysis suggests that risk of recurrence is similar to more proximal PE [9, 19].

Within this context, clinical prognostic scores may be useful in assisting clinical decision-making. Pulmonary embolism severity index (PESI) – a tool stratifying risk for all patients with PE [20] has seen wide use. Two prognostic scores have been recently suggested specifically for patients with cancer and PE: The POMPE-C tool identified patient weight, respiratory rate, O_2_ saturation, heart rate, altered mental status, respiratory distress, unilateral limb swelling and “do not resuscitate” status as predictors of 30-day mortality. The POMPE-C score showed better prognostic accuracy than PESI for patients with active cancer [21]. The RIETE investigators [22] proposed a score utilising age > 80 years, heart rate, hypotension, low body weight, recent immobilisation, and metastatic disease, for predicting 30-day mortality. Both the above prognostic scores relate predominantly to the symptomatic PE setting.

In the present paper, we describe a prospective cohort of patients with active cancer and IPE managed uniformly under a specialised nurse-led service, with an analysis of prognostic factors for early mortality and survival. The development of a dedicated clinical prognostic score predicting early mortality and survival based on the above analysis is presented and discussed.

## Methods

In our department (Queen’s Centre for Oncology and Haematology, Castle Hill Hospital, Cottingham, UK, Hull and East Yorkshire Hospitals NHS Trust) all patients with cancer and IPE are managed uniformly under a nurse-led service since March 2010. The details of the development of this service have been previously published [23]. Patient-reported symptomatology at baseline was recorded through a simple dichotomous questionnaire capturing symptoms relative to this cohort of patients [12, 23]. The incorporated risk-assessment algorithm, which guides hospital admission decisions, is described in a previous publication [23] and has been based on PESI with modifications reflecting our experience regarding the outcome of these patients [24, 25]. For the calculation of the PESI score the “cancer present” variable is considered positive when cancer is measurable or evaluable and negative when not (i.e. in patients undergoing adjuvant treatment). All patients receive Low Molecular-Weight Heparin as per institutional guidelines, following the CLOT [26] regimen for dalteparin, unless clinically contra-indicated, in which case a decision from the attending Oncologist is required. All patients on chemotherapy continue secondary prophylaxis with dalteparin; oral anticoagulants are allowed after the completion of chemotherapy at the treating physician’s discretion. Duration of anticoagulation is at the physician’s discretion, but a minimum of six months is recommended.

The study reported in this manuscript is the result of work that has been classified as an audit. This is a regular undertaking in UK hospitals with the primary goal of maintaining quality standards and identifying areas that need improvement if they fall below accepted standards as set by national or international guidelines. Audits can be retrospective or prospective. As per the NHS Health Research Authority guidelines our study, which can be classified within the audit / service evaluation description, does not require external Research Ethics Committee approval [27]. The NHS Trust governance body which authorises the project is doing so if the study is conducted within the regulatory framework including the Data Protection Act (1998), the Caldicott principles (1997) and the NHS Confidentiality code of practice (2003) [28]. Within this context regulatory approval is sought and obtained based on the quality of the audit and the priority of the area studied and whether it fits within the quality framework of the organization. The endorsement code for our study is No. 2013.287, issued by the Hull and East Yorkshire Hospitals NHS Trust the 29th of November 2013.

Baseline data for all patients referred and treated by the nurse-led service for the management of incidental PE were recorded in real or near real-time in an MS EXCEL®2010 (Microsoft Corp™) spreadsheet maintained in a secure virtual hard-drive with restricted access. Outcome data were collected every six months with the help of the electronic medical record system (iSOFT Patient Centre®, CSC™ and Lorenzo®, CSC™).

The database prospectively collects demographic data, weight, data regarding cancer diagnosis, current or recent cancer treatment, medical history and long-term medication, the presence of central lines, recent (30-day) surgery or hospitalisation, clinical variables included in the pulmonary embolism severity index (PESI) and the site and extent of imaged pulmonary thrombi. Patient-reported symptomatology is stratified into new, stable pre-existing and worsening pre-existing. Laboratory investigations include full blood count, biochemical profile (standard electrolyte, renal and liver function assays) and D-dimer level on diagnosis, platelet count on day 7, and records ECOG/WHO performance status (PS) (Additional file 1: Appendix A) at the time of IPE [29]. Outcomes recorded are mortality and survival, recurrence of VTE, haemorrhage and 30-day hospitalisation.

CT thorax imaging in our centre is typically obtained with a slice thickness of 1 mm. Images are reviewed in in workstations running the Phillips Extended Brilliance Workspace ® software package (currently on version V3.5.35.1011) and the Agfa PACS Impax Workstation ® software package (current version V6.5.2.657). All laboratory haematological and biochemical analyses were performed in the same laboratory and were conducted as per the standard local quality assurance protocols.

The present study analyses patients with active cancer included in the pathway between March 2010 and December 2014. The database was closed in May 31, 2015. Active cancer is defined as cancer present or current cancer treatment (i.e. adjuvant treatment) or treatment for cancer within the past six months.

### Statistical considerations

All analyses were performed with SPSS ver22, IBM Corp ®.

Descriptive statistics were used to analyse patient characteristics. Survival and treatment related outcomes (VTE recurrence and haemorrhage) were calculated from the date of PE diagnosis.

Univariate correlations of continuous variables including age, WCC, Hb, PLT, D-Dimers, with 30-day, 3-month and 6-month mortality were performed with Receiver Operator Characteristics (ROC) curve analysis. ROC analysis was also used to assess cut-off points when needed. Variables which are constituents of the PESI score were not analysed as separate factors.

Survival was calculated from the date of IPE diagnosis until the date of last follow-up contact or the date of death. Only two patients were lost to follow-up and are included in the analysis. The Kaplan Meier method was utilised to explore the prognostic significance of categorical variables using the log rank test to compare factors. Multivariate analyses were performed with the logistic regression method for mortality and the Cox Proportional hazards method for survival. Case-wise exclusion was used in all analyses to handle missing data.

The Kendall tau-b test was utilised to assess correlation of risk categories with mortality event numbers at the 30-day, 3- and 6- month cut-offs.

A probability threshold of 5% was used to define statistical significance in all analyses.

### Prognostic score development

The analytical process for the development of a prognostic score is illustrated in Additional file 1: Appendices B and C of the Supplementary Material. Variables with prognostic significance for early (30-day, 3-month, 6-month) mortality and overall survival were candidate for inclusion in a prognostic score predicting mortality of cancer patients with IPE. The Wald statistic from the Cox Regression analysis was used to weigh the relative prognostic significance of different variables and assign score points to each variable included in the scores. Risk score grouping was performed by assessing the Kaplan-Meier survival curve clustering for different point aggregates. ROC analysis was used to assess the fitness of different prognostic scores.

## Results

234 patients are included in this analysis. Baseline characteristics and assessments of patients are shown in Table 1.

**Table 1:** Characteristics of 234 patients with cancer and IPE

	% (*n*)^+^
Age	
	[Median: 67 (Range: 27–91)]
Gender	
Male	59 (139)
Female	41 (95)
Setting	
Radical/adjuvant	80 (188)
Metastatic/incurable	20 (46)
Diagnosis	
Colorectal cancer, early	5 (12)
Colorectal cancer, metastatic	20 (46)
Oesophagogastric Cancer, early	7 (17)
Oesophagogastric Cancer, metastatic	9 (21)
Breast Cancer, Metastatic	9 (21)
Pancreaticobiliary Cancer, Advanced	9 (21)
NSCLC Metastatic/ SCLC *	12 (28)
Other	29 (68)
Treatment	
Cytotoxic chemotherapy	66 (154)
Biologic/targeted therapy **	13 (30)
Hormonal manipulation therapy***	4 (10)
Interferon	1 (2)
Risk Factors for VTE	
Recent (30d) hospitalisation	15 (36)
Recent (30d) Surgery	2 (5)
Indwelling CVC	15 (35)
PS	
0	45 (105)
1/2	43 (100)
3/4	10 (23)
MD	3 (6)
Extent of IPE	
Bilateral	39 (91)
Largest vessel: pulmonary artery (main, right, left)	20 (46)
Largest vessel: lobar branch(es)	27 (63)
Largest vessel: segmental or subsegmental	42 (99)
Largest vessel: subsegmental branches	11 (25)
Symptoms (self reported)	
Any new symptom	42 (98)
Worsening pre-existing symptoms	21 (49)
PESI group	
I/II	12 (29)
III	42 (99)
IV	37 (86)
V	8 (20)

*Extensive and limited stages, ** CD20, VEGF, EGFR, HER2 - targeted monoclonal antibodies or tyrosine kinase inhibitors., *** Tamoxifen, aromatase inhibitors, antiandrogen, GnRH

+ percentages rounded for simplicity, may not add up to 100

### Symptoms

Symptoms recorded included: dyspnoea (*n* = 121 51.7%), fatigue (*n* = 181, 77.4), chest pain (*n* = 26, 11.1%), lower limb oedema (*n* = 78, 33.3%), haemoptysis (*n* = 8, 3.4%). Lower limb Doppler U/S was not required as part of the work-up, nevertheless 16 patients had documented concurrent DVT. Overall 121 patients reported new or worsening pre-existing symptoms (52%).

### Outcomes

Median follow-up for patients remaining alive at study closure was 36.7 months, 95%CI (27.5, 45.9).

#### Survival, mortality

72 patients were alive at the time of database closure; 2 patients were lost to follow-up. 30-day, 3-month and 6-month mortality was 3.4% (*n* = 8), 15% (*n* = 35) and 30.7% (*n* = 72) respectively. Median overall survival (OS) for the entire cohort was 12.6 months 95%CI (9.4, 15,8).

In ROC analysis WCC demonstrated a correlation with 30-day (*p* = .005), 3- and 6- month mortality (*p* = .001 and *p* < .001 respectively) whilst serum Creatinine demonstrated a (negative) correlation with 3- and 6-month mortality (p < .001 and *p* = .002), but it was not predictive of 30-day mortality (*p* = .100). Age, PLT and D-Dimer levels showed no correlation to mortality. Multivariate logistic regression analysis of clinical and laboratory factors with potential prognostic significance as selected by univariate analysis is shown in Table 2. Survival analysis indicated palliative (non-curative) setting, new and worsening symptoms (individually or combined), PS and PESI as eligible prognostic factors (Table 3).

**Table 2:** Logistic regression analysis

	30-d mortality *n* events: 7/219**		3-month mortality *n* events: 32/219**		6-month mortality *n* events: 64/219**	
Category	OR (95%CI)	*p*	OR (95%CI)	*p*	OR (95%CI)	*p*
Palliative setting (metastatic or incurable malignancy)	NC	.998	1.6 (.3, 1.7)	.596	1.6 (.6,4.6)	.384
PS		.239		.050		< .001
PS 0	1		1		1	
PS 1,2	1.6 (.1, 20.9)	.719	1.3 (.5, 3.7)	.932	2.7 (1.3, 5.9)	.010
PS 3,4	6.4 (.1, 87.6)	.163	4.8 (1.3, 17.9)	.019	13.7 (4, 47.2)	< .001
PESI		> .999		.096		.142
PESI Class I/II	1		1		1	
PESI III/IV	NC	.998	4.1 (.7, 22.6)	.107	0.97 (.4, 2.7)	.943
PESI V	0.7 (NC)	> .999	11.7 (1.3, 101.8)	.030	3.3 (0.7, 15.4)	.126
New/worsening symptoms	4 (.4, 40.2)	.239	1.9 (.8, 4.8)	.159	2.9 (1.4, 6)	.005
WCC (cont.) *	1.3 (1.1, 1.6)	.013	1.2 (1, 1.3)	.008	1.1 (1, 1.3)	.020
Creat (cont.) *	.98 (.9, 1)	.372	.9 (.9, .98)	<.001	.98 (.96, .99)	.014

Odds ratios are rounded to the first decimal (except when too close to 1) for simplicity. *NC* not computed. (cont.)*: continuous variable. **: number of patients with complete data in all categories

**Table 3:** Univariate (Kaplan-Meier) and multivariate (Cox Regression) analysis for overall survival.

	N*	OS (95%CI)	p	HR(95%CI)	p
Metastatic/incurable disease					
No	45	NR 10.5 (7.3, 13.7)	<.001	1	<.001
Yes	188			2.6 (1.5, 4.3)	
New Symptoms					
No	132	17 (12.7, 21.3)	.029		
Yes	98	10.1 (5.9, 14.3)			
Worsening Symptoms					
No	178	15.2 (11.9, 8.4)			
Yes	49	6.5 (5.2, 7.8)	.013		
New or Worsening symptoms					
No	111	19 (14.3, 23.8)	.004	1	.002
Yes	121	8.6 (5.3, 11.8)		1.7 (1.2, 2.4)	
WCC				1	
<11.3 x10^9^/L	199	14 (9.9, 18.1)	.016	1.1 (.7, 1.8)	
≥11.3 x10^9^/L	26	5.4 (2.4,8.3)			.659
Creatinine					
<55μmol/L	29	5.3 (1.5, 9.2)	.021	1	.408
>55μmol/L	195	14 (10, 17.9)		1.2 (.8, 2)	
ECOG/WHO PS			<.001		<.001
0	104	22.9 (17.9, 28.3)		1	
1,2	100	8.8 (6.5, 11)	<.001	1.9 (1.3, 2.8)	.001
3,4	23	3.3 (2.2, 4.4)	<.001	3.7 (2.2, 6.3)	<.001
PESI			<.001		.081
I,II	28	21 (6.2, 35.7)		1	
III,IV	185	13.8 (10.5, 17.1)	.222	1.5 (.8, 2.5)	.181
V	20	5.5 (4.3, 6.6)	<.001	2.3 (1.1, 4.8)	.026

Numbers are rounded to the first decimal for simplicity were possible. ROC curve analyses were used to identify candidate cut-offs for dichotomisation of continuous variable, indicating WCC 11.3 x10^9^/L and Creatinine 55umol/L as useful cut-offs. 219 complete cases with 152 events available for Cox Regression

A significant correlation of increasing WCC levels, as continuous variable, with early mortality in both univariate as well as multivariate models was observed. ROC analysis indicated a cut-off of WCC = 11.3 × 10^9^/L as suitable for dichotomisation. Patients with WCC > 11.3 × 10^9^/L had 30-day mortality rate of 15.4% versus 2% for patients with WCC < 11.3 × 10^9^/L (*p* = .001). The same cut-off appeared to correlate with overall survival (OS) (*p* = .016). Decreasing creatinine levels also showcased a relation to 3-month and 6-month mortality with a cut-off of <55 μmol/L identified as appropriate for dichotomisation in ROC analysis and in univariate survival analysis this cut-off showed a significant correlation with OS (*p* = .0210).

The distribution of PE was analysed as an ordinal categorical variable, recoded as central vs others, as subsegmental vs others with and without corrections for bilaterality. No significant effects on survival were observed.

Factors demonstrating significance in univariate survival analysis were entered in a multivariate survival model as shown in Table 3. Creatinine, WCC levels and PESI score did not retain prognostic significance.

#### Recurrence

In total, 20 recurrent or progressive VTE events were recorded within the follow up period [rate: 8.5%, 95%CI (5.1%, 12%)]. VTE recurrence/progression rate in the first three months was 0.9% (*n* = 2) 95%CI (0,2%, 1%), and within the first six months 2.6% (*n* = 6) 95%CI (0.9%, 4.7%). In 6 cases (2.6%) recurrent VTE occurred while on thromboprophylaxis. In one case DVT occurred in a post-operative period while on prophylactic dose of dalteparin and with an IVC filter in situ. One of the patients was on warfarin. Median time to recurrent/progressive VTE was 9.6 months 95%CI (8.5, 10.8).

#### Haemorrhage

13 haemorrhagic complications were recorded [5.5%, 95%CI (3%, 8.5%)] at a median of 3 months 95%CI (1.3, 4.7) from the time of IPE. Major haemorrhage as per the ISTH criteria (>2.0 g/L drop in Hb, fatal, or haemorrhage in critical area) was represented with 5 cases for an incidence of 2.1%, 95%CI (0.4%, 3.8%) and occurred at a median of 3.3 months 95%CI (1.5, 5.1). Major haemorrhage within six months occurred in 4 patients [1.7% 95%CI (.4%, 3.4%)].

#### Hospitalisation

23 patients in this cohort were admitted to the hospital as per protocol (9.8%). Four patients (1.7%) were inpatients at the time of IPE diagnosis. 34 patients amongst the 207 who were managed as out patients (16%) were hospitalised within 30 days of IPE. The cause for hospitalisation was recorded as “bleeding” in two (1%) and “pulmonary embolism” in two patients (1%).

#### Prognostic factors for haemorrhage, recurrence and 30-day hospitalisation

Multivariate logistic regression analyses were performed with clinical and laboratory factors for haemorrhage, major haemorrhage, recurrence of VTE and hospitalisation within 30 days. In these analyses PESI score class V was the only factor showing a trend for correlation with 30-day hospitalisation (*p* = .0520), HR 8.45 95%CI (.98, 72.8). Increasing creatinine levels were associated with all-grade haemorrhage (*p* = .010), HR 1.03 95%CI (1.01, 1.05), but no factor was associated with major haemorrhage. Cox regression suggested a HR of 5.40 95%CI (1.71, 17.04) for haemorrhage for Creatinine levels with a cut off of 108.5 μmol/L (>90% specificity in ROC curve analysis). No factor was associated with VTE recurrence or VTE recurrence within six months.

#### Prognostic score for mortality

We have previously described a feasible prognostic score for patients with cancer and IPE based on the presence of new symptoms, PS and the presence of incurable cancer, constructed on a subset of the current dataset [30]. Compared with our previous analysis, in the current cohort, PESI score class V appeared to convey an independent survival detriment compared to the reference category (PESI I/II) both in regards 3- month mortality (*p* = .030) as well as in terms of OS (*p* = .026) (Tables 2,3). On the other hand, the effect of the overall PESI variable remained non-significant on survival (*p* = .081) as well as on 30-day, 3-month and 6-month mortality (*p* > .999, *p* = .096 and *p* = .142 respectively) (Tables 2,3).

The RIETE score was also applied to this dataset. The POMPE-C score could not be considered due to missing “do not resuscitate” information. For the purposes of our analysis, hospitalisation within the past 30 days and surgery during the past 30 days were used to derive the “recent immobilisation” category of RIETE, since this was not a variable in our data recording as such.

To evaluate the feasibility of a clinical prognostic score for cancer patients with IPE deriving from our cohort we used information from logistic regression analysis on 30-day, 3-month and 6- month mortality as well as from multivariate survival analysis as shown in Tables 2 and 3. The most consistent predictors of survival in these analyses were the patient-reported symptomatology (new or worsening) and performance status at the time of IPE diagnosis. The presence of metastatic-incurable cancer had strong association with OS but not with early mortality (Tables 2 and 3). Utilising the Wald statistic for weighing significant variables and Kaplan-Meier curves for the grouping of categories, four possible scoring systems were identified; three included (a) the presence of metastatic disease, (b) the presence of new or worsening symptoms at the time of IPE diagnosis and (c) Performance status (0 vs 1,2 vs 3,4). The fourth scheme (“Hull5”) excluded variable (a) since it appeared to lack association with early mortality (Additional file 1: Appendix Β). All four scores were compared with ROC analysis against RIETE and PESI with all scores initially treated as continuous variables and subsequently grouped in prognostic categories (Additional file 1: Appendix C). PESI did not achieve significance in any of these analyses. The RIETE score showed an association with 3-month and 6-month mortality. The RIETE very low risk, low risk, intermediate and high risk categories retained correlation with 3- and 6- month mortality, but the HIGH/LOW grouping [22] did not. In all ROC curve analyses the Hull clinical scores appeared to outperform RIETE groups, except for 3-month mortality (Additional file 1: Appendix C).

**Fig. 1:**
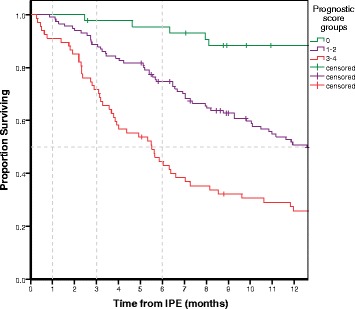
Survival curves for the Hull5 score groups for the first 12 months of follow-up. Line separators for the 30-day, 3-month, 6-month cut-offs and the median for survival are included

Two of the experimental scores exhibited greater predictive consistency (Additional file 1: Appendix C); one included all three clinical variables (a), (b) and (c) as above (“HULL2”) and the other only (b) and (c) (“HULL5”). “HULL2” resulted in four risk groups identified by clustering of Kaplan-Meier survival curves. “HULL5” (Table 4) produced three risk clusters in Kaplan-Meier (low, intermediate, high risk, Fig. 1). Both these scores exhibited significant predictive ability for early mortality [30-day mortality: AUC = .821, 95%CI (.707, .936), *p* = .004 and AUC = .805, 95%CI (.675, .934), *p* = 0.006, respectively, as seen in Additional file 1: Appendix C]. An attempt to analyse “HULL2” grouped into three prognostic categories resulted in loss of significance in ROC analysis, therefore it was discarded in favour of “HULL5”. We would like to note, though, that the initial significance of “HULL2”, does demonstrate that asymptomatic/good PS patients treated for potentially curable malignancy can be considered a particularly favourable prognostic category. Table 5 illustrates the OS and mortality rates of prognostic groups in our patient cohort as per the “HULL5” score system in comparison to the RIETE score prognostic grouping.

**Table 4:** Derivation of the Hull5 prognostic score from a multivariate Cox regression model for OS with two selected variables exhibiting association with OS and early mortality

Variable	Categories	Wald	HR	95%(CI)	*P*	Points
New or worsening symptoms	Yes	10.962	1.7	(1.3, 2.4)	.001	1
	No	1	1			0
Performance status	0	1	1			0
	1/2	18.33	2.1	(1.5, 3)	< .001	2
	3/4	28.2	3.9	(2.3,6.3)	< .001	3

Grouping - Low Risk: 0, Intermediate Risk: 1–2, High Risk: 3–4

**Table 5:** OS, 30-day, 3-month and 6-month mortality of discussed risk scoring

Score categories	OS months (95%CI)	Mortality %(*n*)		
		30-d (*n* = 7)	3-m (*n* = 33)	6-m (*n* = 68)
Hull5 Risk Score (*n* = 227)				
Low (*n* = 45)	32(8.1,5 5.9)	0(0)	2.2(1)	4.4(2)
Intermediate (*n* = 115)	12.6(8.3, 16.9)	0.9(1)	11.3(13)	25.2(29)
High (*n* = 67)	5.5 (3.9, 7.2)	9.0(6)	28.4(19)	55.2(37)
	p < .001*	p = .004**	p < .001**	p < .001**
RIETE Risk Score (*n* = 205)				
Very low (*n* = 27)	*not reached*	0(0)	3.7(1)	7.4(2)
Low (*n* = 123)	15.9 (11.2, 20.6)	2.4(3)	9.8(12)	22.8(28)
Intermediate (*n* = 53)	5.4 (4.3, 6.5)	5.7(3)	32.1(17)	56.6(30)
High (*n* = 2)	2.7 (not calc.)	0%(0)	50(1)	50(1)
	p < .001*	p = .185**	p < .001**	p < .001**
RIETE Risk Score (*n* = 205)				
Low(n = 27)	*not reached*	0(0)	3.7(1)	7.4(2)
High (*n* = 178)	11.2 (7.7, 14.7)	3.4(6)	16.9(30)	33.1(59)
	p < .001*	p = .603**	p = .087**	p = .006**

*log rank (pooled)

**Kendall’s tau-b exact test

## Discussion

The main strength of the current analysis is that it derives from a prospectively identified cohort managed uniformly under a standardised diagnostic and management protocol developed and applied in real-life conditions in a single centre. The generalisability of our observations would require validation in an external cohort.

6-month mortality in our cohort was 30.8% 95%CI (24.8, 36.8), similar, though numerically lower, to the mortality rate reported in the largest published pooled cohort of cancer patients with IPE [37% 95% CI (28%, 47%)] [19]. 30-day mortality in the cohort of 408 patients used for the derivation of the POMPE-C score was 12.5% (15% in their external validation cohort of 182 patients) and 21% in 508 patients used for the derivation of the RIETE prognostic score (24% in the external validation cohort of 261pts) [22]; both of these studies included patients with active cancer and predominantly symptomatic PE. In our cohort 30-day mortality was 3.4% 95%CI (1.3%,6,0%) possibly reflecting a difference in the outcomes of ambulatory IPE versus acute symptomatic PE patients with active cancer.

Recurrence of VTE or progressive PE within six months as well as major haemorrhage within six months were rare in our patients, comparing favourably to the report by vanderHulle et al. [19]. The reason for this difference is unclear, but it should be noted that our cohort was treated under a specific standardised protocol for treatment initiation and follow-up.

This analysis verifies the observations we previously made in a subset of this cohort suggesting that the usefulness of the standard PESI score may have reduced value in this group of patients [30]. In the current analysis, PESI class V did demonstrate statistically significant effects on mortality, as expected, since it describes patients with significant comorbidities and/or evidence of physiological compromise, nevertheless in ROC analysis PESI scoring as a continuous variable was the weakest amongst the tested risk scoring algorithms. RIETE performed better in our cohort still less consistently than the proposed clinical score. It is therefore our observation that a basic oncologic assessment of performance status at the time of IPE, combined with the self-reported presence of new or worsening symptomatology (without further elaboration) may reliably risk-stratify cancer patients with an incidental finding of pulmonary embolus.

Our analyses identify a group of patients with particularly good prognosis, namely those with an excellent prognostic status, absence of new or worsening symptoms which may be further enriched by excluding patients with metastatic disease. For this group of patients, modification of the standard management approach, for example reducing the duration of anticoagulation treatment, may be considered, still such a recommendation would require a randomised study.

Two prognostic scores have been proposed in the literature for patients with cancer and acute pulmonary embolism each utilising multiple clinical and laboratory parameters. The POMPE-C score [21] incorporates eight clinical variables and RIETE [22] six. The RIETE investigators also identified two additional laboratory variables namely WBC > 11,000/mm^3^ and Creatinine clearance <30 ml/min. The prognostic significance of WCC was observed in our analysis regarding early mortality, nevertheless its significance was lost in multivariate OS analysis. A minor effect of serum creatinine was also observed nevertheless this effect was inverse to the RIETE observations. One interpretation of our observation could be that very low creatinine levels may correlate with cancer cachexia which itself may convey worse prognosis. We did not include WCC or Creatinine levels in the prognostic score analyses because the effects on OS were not maintained in the multivariate Cox regression model but also because their effect was relatively small as indicated with OR close to the unit and since a purely clinical prognostic score may be more readily applicable and interpretable.

Our application of RIETE in this analysis used the recorded variables of hospitalisation in the previous 30 days and surgery in the previous 30 days as surrogates for the immobilisation variable. This approximation may have introduced bias in our comparative analysis nevertheless it may also be regarded as a showcase of the inherent subjectivity of this variable. We were not able to apply POMPE-C since we did not have the information regarding Do-Not-Resuscitate orders, which in addition, is a non-standardisable variable which introduces bias. It is our view that the assessment of performance status and the self-reported presence of new or worsening symptomatology is a simple and generalizable assessment. The presence of incurable/metastatic cancer, which may enrich the risk assessment, is also simple and objective.

Symptomatology in our analysis was a self-reported variable and did not require further elaboration to achieve prognostic significance. This observation may be considered in concert with previous publications [12]. The presence of new symptomatology and the deterioration of pre-existing symptoms exhibited a significant independent correlation with survival which may illustrate the effect of the unsuspected pulmonary embolus in patient physiology, or possibly the development of PE within the context of progressive malignancy which may cause deteriorating symptoms and also carries prothrombotic potential.

As limitations of the current work we can consider the lack of granularity of patient-reported symptom assessment as well as the lack of follow-up of symptomatology which could provide a prognostic assessment of patient-reported outcomes (PRO). We are currently piloting the utilisation of validated symptom-scale questionnaires (i.e. SF12 and PembQUOL) and plan to study patient-reported outcomes within this patient group. It should also be noted that our cohort, similarly to previously published studies, was not arithmetically powered for cancer-specific sub analyses (exploratory analyses are shown in Additional file 1: Appendix D). It is conceivable that different malignancies provide a different level of competing risk for early mortality. In addition, the relatively small number of haemorrhages, VTE recurrences and 30-day hospitalisations observed in our study population may limit the accuracy of our corresponding prognostic analysis; more accurate observations would require the analysis of these data points in larger cohorts. Moreover, since our study was based on audit data, it analysed only the baseline factors that were available as part of the standard clinical and laboratory assessments required for the safe management of the patients within our clinical pathway. Therefore, factors such as smoking history and mean platelet volume (MPV) which are known potential prognostic factors for the development of VTE or additional experimental baseline characteristics were not recorded or analysed.

## Conclusion

In conclusion, our analysis suggests that a simple prognostic score based on the patient reported clinical factors -symptom assessment and contemporaneously assessed performance status - reflecting the physiological burden on a cancer patient - can be used to easily and reliably stratify the mortality outcomes of patients with IPE and cancer in the clinical setting.

## Additional file

Additional file 1: Appendices. (DOCX 493 kb)

## Abbreviations

CLOT: Comparison of Low-molecular-weight heparin versus Oral Anticoagulant Therapy for the prevention of recurrent venous thromboembolism in patients with cancer (clinical trial acronym)
CT: Computed Tomography
DVT: Deep Vein Thrombosis
ECOG: Eastern Cooperative Oncology Group
Hb: Haemoglobin
IPE: Incidental / Unsuspected Pulmonary Embolism
ISTH: International Society for Thrombosis and Haemostasis
IVC: Inferior Vena Cava
LMWH: Low Molecular Weight Heparin
OS: Overall Survival
PE: Pulmonary Embolism
PESI: Pulmonary Embolism Severity Index
PLT: Platelet count
PS: Performance Status
RIETE: Registro Informatizado de Pacientes con Enfermedad TromboEmbólica (Computerised Registry of Patients with Venous Thromboembolism)
ROC: Receiver Operator Characteristics
VTE: Vascular Thromboembolic Events
WCC: White Cell Count
WHO: World Health Organisation
