# Correction to: Prognostic assessment for patients with cancer and incidental pulmonary embolism

**DOI:** 10.1186/s12959-018-0169-1

**Published:** 2018-04-18

**Authors:** George Bozas, Natalie Jeffery, Deiva Ramanujam-Venkatachala, Ged Avery, Andrew Stephens, Hilary Moss, June Palmer, Mandi Elliott, Anthony Maraveyas

**Affiliations:** 1 Hull and East Yorkshire NHS Hospitals Trust, Queen’s Centre for Oncology and Haematology, Castle Hill Hospital, Castle Road, Cottingham, HU16 5JQ UK; 2 Hull and East Yorkshire NHS Hospitals Trust, Radiology Department, Castle Hill Hospital, Castle Road, Cottingham, HU16 5JQ UK; 3 Hull York Medical School, Academic Oncology, Castle Hill Hospital, Castle Road, Cottingham, HU16 5JQ UK

## Correction

Following the publication of this study [1] the authors identified the following errors inadvertently introduced during the production process:

1. Within Table 1, under the sub-heading “Setting”, the numbers for the categories “Radical/adjuvant” and “Metastatic/incurable” were erroneously inversed. The correct numbers are:
  Radical/adjuvant: 20 (46)
  Metastatic/incurable: 80 (188)
2. In Table 3, the values in the HR(95%CI) column were misaligned in the WCC rows; The value “1” should be aligned with the <11.3x10^9^ row and the value 1.1 (.7, 1.8) should be aligned with the >11.3x10^9^ row.
3. Also in Table 3, within the OS (95%CI) column and under the subheading “Metastatic/incurable disease”, the value “10.5 (7.3, 13.7)” was misaligned and should be aligned with the “Yes” row.
  Tables 1 and 3 have now been updated in the original article.
4. In the “Acknowledgements” section, the Fig. 1 legend “Survival curves for the Hull5 score groups for the first 12 months of follow-up. Line separators for the 30-day, 3-month, 6-month cut-offs and the median for survival are included”, was inserted in error.

Furthermore, for additional clarity, where the term “Vascular Thromboembolic Event” is used in the text, the reader is advised to read “Venous Thromboembolic Event” **.**
