# Supplementary material for: Prognostic assessment for patients with cancer and incidental pulmonary embolism
Source: Thromb J. 2018 Feb 6;16:8. doi: 10.1186/s12959-017-0157-x (PMC5802055; doi:10.1186/s12959-017-0157-x)
Supplement: Additional file 1: — Appendices. (DOCX 493 kb) [file 12959_2017_157_MOESM1_ESM.docx]

# Prognostic assessment for patients with cancer and incidental pulmonary embolism.

Georgios Bozas1, Natalie Jeffery1, Deiva Ramanujam-Venkatachala1, Ged Avery2, Andrew Stephens2, Hillary Moss1, June Palmer1, Mandi Eliott1, Anthony Maraveyas1,3

1 Hull and East Yorkshire NHS Hospitals Trust, Queen’s Centre for Oncology and Haematology, Castle Hill Hospital, Cottingham, UK

2 Hull and East Yorkshire NHS Hospitals Trust, Radiology Department, Castle Hill Hospital, Cottingham, UK

3 Hull York Medical School, Hull, UK

# Appendix A

**Table 1.** Easter Cooperative Oncology Group / Whorld Health Organisation Performance Status classification (also referred to as Zubrod scale). Abbreviation: ECOG/WHO PS.

Referrence: Oken M, Creech R, Tormey D, et al. Toxicity and response criteria of the Eastern Cooperative Oncology Group. Am J Clin Oncol. 1982;5:649-655.

| PS | Description |
| --- | --- |
| 0 | Fully active, able to carry on all pre-disease performance without restriction |
| 1 | Restricted in physically strenuous activity but ambulatory and able to carry out work of a light or sedentary nature, e.g., light house work, office work |
| 2 | Ambulatory and capable of all selfcare but unable to carry out any work activities; up and about more than 50% of waking hours |
| 3 | Capable of only limited selfcare; confined to bed or chair more than 50% of waking hours |
| 4 | Completely disabled; cannot carry on any selfcare; totally confined to bed or chair |
| 5 | Dead |

# Appendix B

Four candidate scores where investigated in this analysis. Appendix A details the derivation of these scores.

- Hull1 score derived from the comparison of the Wald statistics in the Cox Regression model of statistically significant variables in the multivariate analysis as presented in the manuscript in Table 3.
- Hull2 is a simplification of the scoring of Hull1 score
- Hull3 derives from the comparison of the Wald statistic in a Cox Regression model which included only the previously identified significant variables (a) New/Worsening Symptoms, b) Metastatic/Incurable solid tumour, c)Performance status)
- Hull5 derives from the comparison of the Wald Statistic of a Cox model including only a) New/Worsening symptoms and b) Performance Status. This choice resulted from the observation that Metastatic/ Incurable disease did not have a significant effect on early mortality.
- Please note that an exploratory attempt for a Hull4 score was abandoned due to a coding error and was therefore not considered and is not presented here.

### HULL1 Candidate Score

**Table 2**. Hull1 Derivation Multivariate analysis output (Cox Regression)

| **Variables** | **Categories** | **Wald** | **HR** | **(95.0%)** | | **p** | **Points assigned** |
| --- | --- | --- | --- | --- | --- | --- | --- |
| Palliative setting (metastatic or incurable disease) | Yes No | 12.218 | 2.55 1 | (1.51, 4.31) | |  | 3 0 |
| New or worsening symptoms | Yes No | 9.613 | 1.69 1 | (1.21, 2.36) | | .000 | 2 0 |
| WCC_REC_113 | <11.3x10^9^/L >11.3x10^9^/L | .195 | 1 1.11 | (.69, 1.8) | | .002 |  |
| CREAT_55 | <55μmol/L >55μmol/L | .684 | 1.23 1 | (.76, 2) | | .659 |  |
| Performance status | 0 | 26.124 | 1 |  |  |  | 0 |
|  | 1/2 | 12.001 | 1.92 | (1.33, 2.8) | | .000 | 3 |
|  | 3/4 | 23.522 | 3.7 | (2.18, 6.29) | | .001 | 6 |
| PESI categories | I/II | 5.033 | 1 |  |  |  |  |
|  | III/IV | 1.787 | 1.46 | (.84, 2.53) | | .181 |  |
|  | V | 4.973 | 2.31 | (1.11, 4.82) | | .026 |  |
| Score Grouping (as per Kaplan Meir curves clustering showed in Figure 1): 0 vs 2-3, vs 5 vs >5 | | | | | | | |

**Figure 1.** Kaplan Meier overall survival curves for patients included in the analyses as per Hull1 scores.


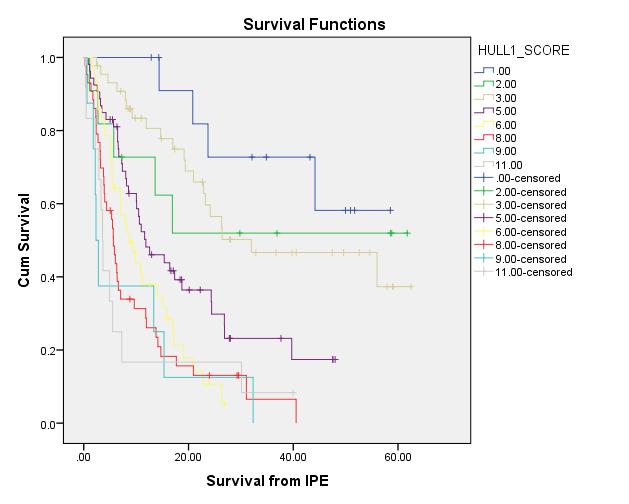


**Figure 2.** Kaplan Meier survival curves for Hull1 score groups (score 0 versus score 2-3, versus score 5 versus score >5).


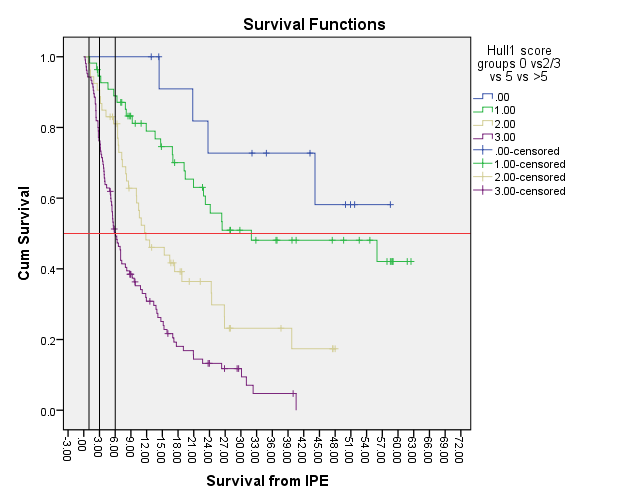


### HULL2 Candidate Score

**Table 3**. Hull2 score utilises the same multivariate analysis as Hull1analysis but simplifies scoring as follows

| **Variable** | **Score** |
| --- | --- |
| Metastatic or incurable disease: Yes  Metastatic or incurable disease No | 1  0 |
| New or worsening symptoms: Yes  New or worsening symptoms: No | 1  0 |
| PS: 0  PS: 1/2  PS: 3/4 | 0  1  2 |
| Score Grouping as per Kaplan Meir curves clustering (Figure 3): 0 vs 1 vs 2 vs 3-4 |  |

**Figure 3**. Kaplan Meier overall survival curves for Hull2 scores.


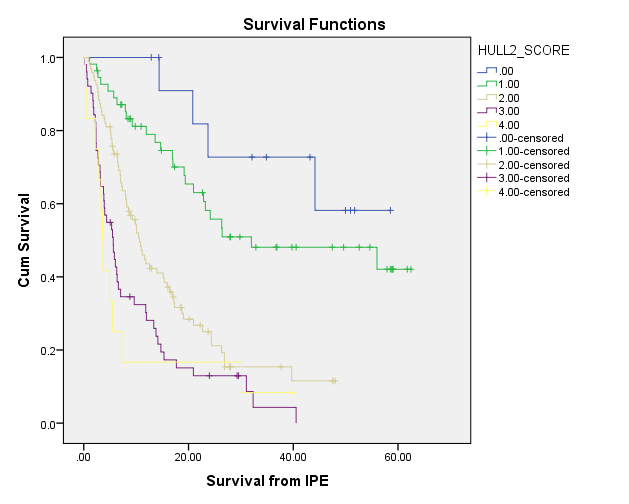


**Figure 4.** Hull2 score groups (score 0 versus score 1 versus 2 versus score 3-4) as per KM curve clustering


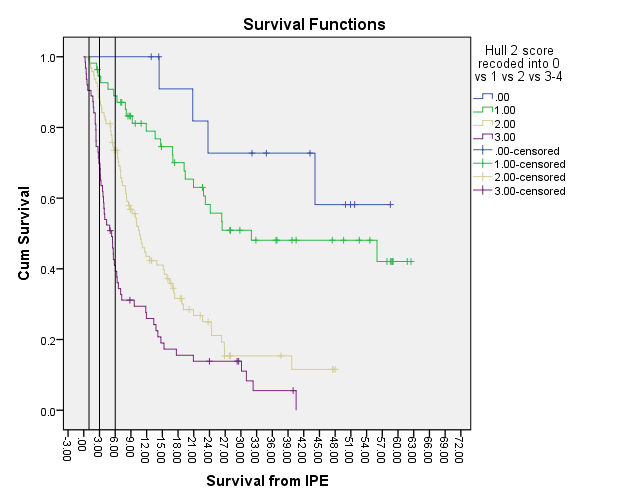


###

### HULL 3 Candidate score

**Table 4.** Hull3 score derivation analysis (Cox regression) and grouping

| **Variable** | **Categories** | **Wald** | **HR** | **95%(CI)** | **P** | **Points assigned** |
| --- | --- | --- | --- | --- | --- | --- |
| Palliative setting (metastatic or incurable disease) | Yes No | 14.819 | 2.67 1 | (1.62, 4.4) | <.001 | 2 0 |
| New or worsening symptoms | Yes No | 6.607 | 1.53 1 | (1.11, 2.12) | .010 | 1 0 |
| Performance status | 0 1/2 3/4 | 18.451 22.644 | 1 2.14 3.36 | (1.51, 3.04) (2.04, 5.53) | <.001 <.001 | 0 3 4 |
| Grouping as per Kaplan Meir Curve clustering (Figure 5): 0 vs 1-2 vs 3 vs >3. | | | | | | |

**Figure 5**. Kaplan Meier curves for overall survival for Hull3 scores


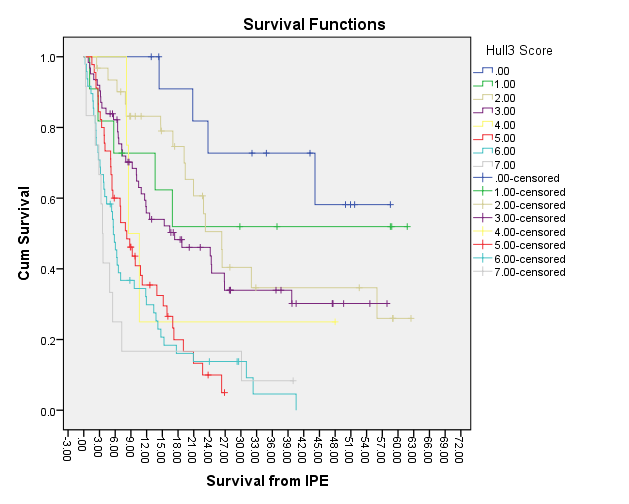


**Figure 6**. Kaplan-Meier curves for overall survival for Hull3 groups.


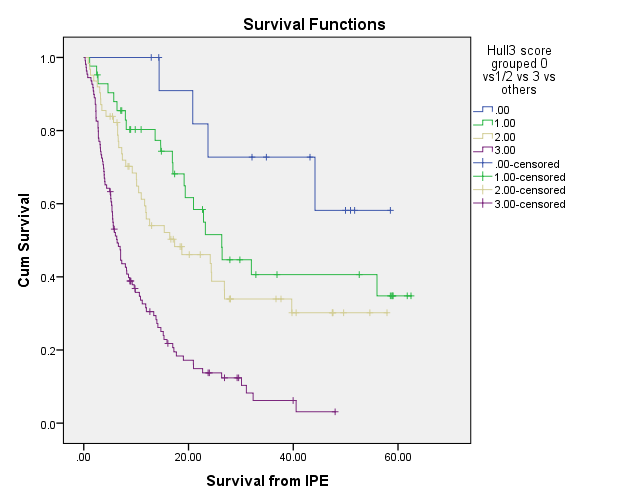


### Hull5 Candidate score

**Table 5.** Hull5 score Derivation from Cox Regression analysis with the two variables with stronger relation with both early and long term mortality (Table 4 in the Manuscript)

| Variable | Categories | Wald | HR | 95%(CI) | P | Points assigned |
| --- | --- | --- | --- | --- | --- | --- |
| New or worsening symptoms | Yes No | 10.962 1 | 1.73 1 | (1.25,2.39) | .001 | 1 0 |
| Performance status | 0 1/2 3/4 | 1 18.33 28.2 | 1 2.14 3.85 | (1.51,3.02) (2.34,6.33) | <.001 <.001 | 0 2 3 |
| Grouping | Low Risk: 0, Intermediate Risk: 1-2, High Risk: 3-4 | | | | | |

**Figure 7.** Kaplan Meier curves for overall survival for Hull5 scores


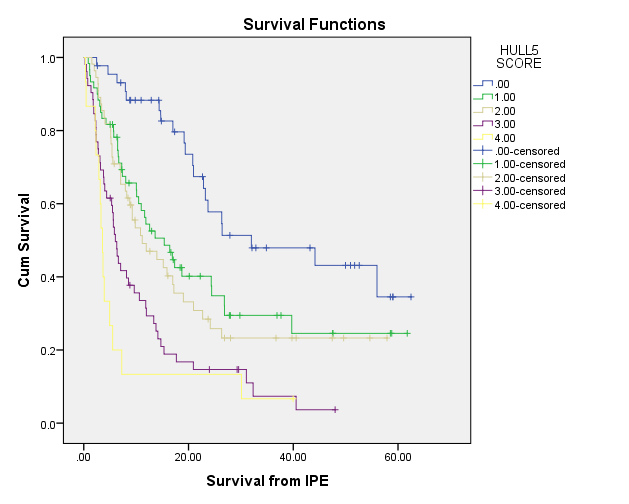


**Figure 8**. Kaplan Meier curves for overall survival for Hull5 score groups. (Figure 1 in the manuscript zooms in the first 12 months of this chart)


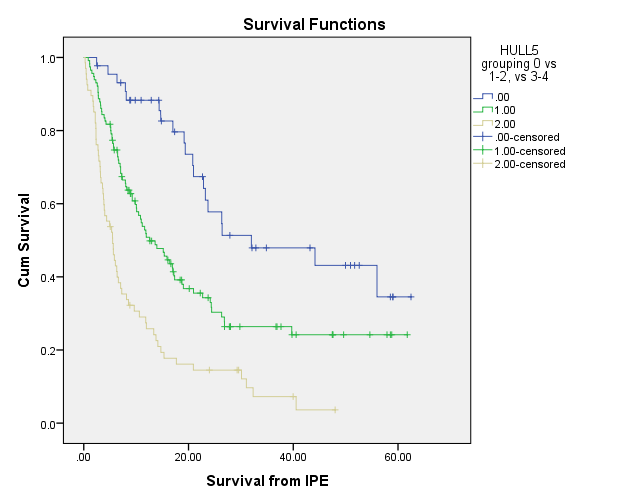


# Appendix C

Receiver Operator Characteristics (ROC) analyses of fitness of studied prognostic scores.

AUC: Area Under the Curve

**Table 6.** Mortality ROC curve comparison of Hull exploratory scores against RIETE and PESI. Scores not grouped.

| **30-day mortality** | |
| --- | --- |
| 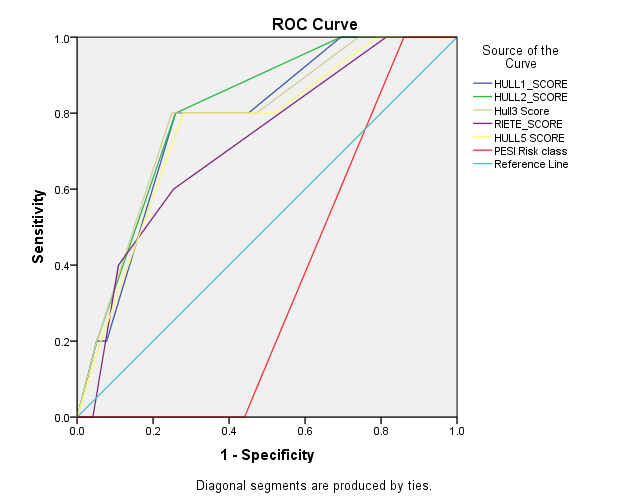 | \| Test Result Variable(s) \| AUC \| P (asympt.) \| \| --- \| --- \| --- \| \| \| HULL1_SCORE \| .779 \| .033 \| \| HULL2_SCORE \| .806 \| .019 \| \| Hull3 Score \| .783 \| .031 \| \| HULL5 SCORE \| .761 \| .047 \| \| RIETE_SCORE \| .720 \| .093 \| \| PESI Risk class \| .350 \| .252 \|   30-day Mortality Valid N (Listwise)  Positive 5  Negative 193  Missing 36 |
| **3-month mortality** | |
| 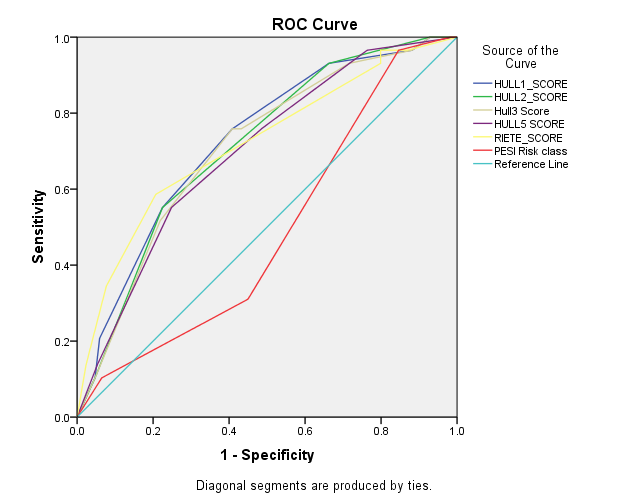 | \| Test Result Variable(s) \| AUC \| P (asympt.) \| \| --- \| --- \| --- \| \| \| HULL1_SCORE \| .727 \| .000 \| \| HULL2_SCORE \| .713 \| .000 \| \| Hull3 Score \| .709 \| .000 \| \| HULL5 SCORE \| .698 \| .001 \| \| RIETE_SCORE \| .720 \| .000 \| \| PESI Risk class \| .487 \| .828 \|   3-month mortality Valid N (listwise)  Positive 29  Negative 169  Missing 36 |
| **6-month mortality** | |
| 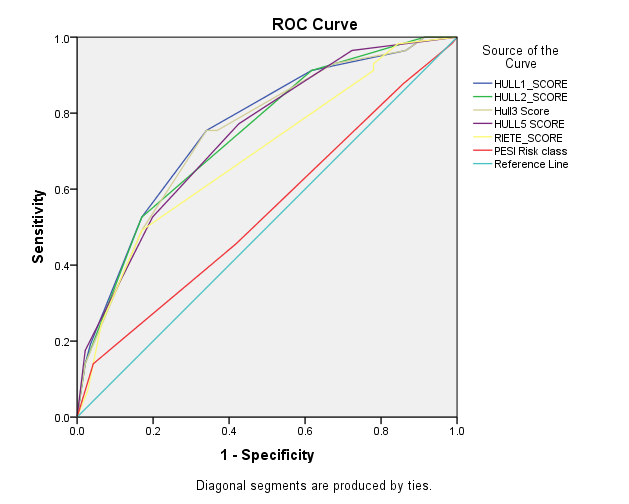 | \| Test Result Variable(s) \| AUC \| P (asympt.) \| \| --- \| --- \| --- \| \| \| HULL1_SCORE \| .759 \| .000 \| \| HULL2_SCORE \| .742 \| .000 \| \| Hull3 Score \| .748 \| .000 \| \| HULL5 SCORE \| .742 \| .000 \| \| RIETE_SCORE \| .689 \| .000 \| \| PESI Risk class \| .541 \| .367 \|   6-month mortality Valid N (listwise)  Positive 57  Negative 141  Missing 36 |

**Table 7.** Mortality Curve Comparisons with score groupings. Two alternative score groupings for Hull3 score are included in the comparison.

| 30-day mortality |  |
| --- | --- |
| 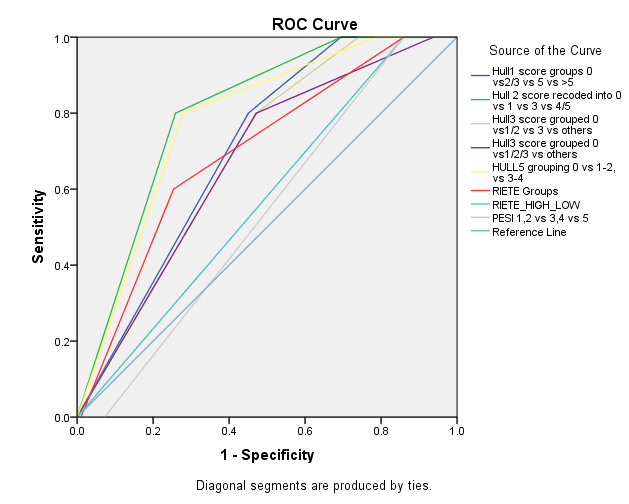 | \| Test Result Variable(s) \| AUC \| P (Asympt.) \| \| --- \| --- \| --- \| \| \| Hull1 score groups 0 vs2/3 vs 5 vs >5 \| .705 \| .118 \| \| Hull 2 score recoded into 0 vs 1 vs 3 vs 4/5 \| .801 \| .022 \| \| Hull3 score grouped 0 vs1/2 vs 3 vs others \| .690 \| .147 \| \| Hull3 score grouped 0 vs1/2/3 vs others \| .670 \| .193 \| \| HULL5 grouping 0 vs 1-2, vs 3-4 \| .781 \| .032 \| \| RIETE Groups \| .698 \| .131 \| \| RIETE_HIGH_LOW \| .570 \| .594 \| \| PESI 1,2 vs 3,4 vs 5 \| .534 \| .797 \|   30-day Mortality Valid N (listwise)  Positive 5  Negative 193  Missing 36 |
| 3-month mortality |  |
| 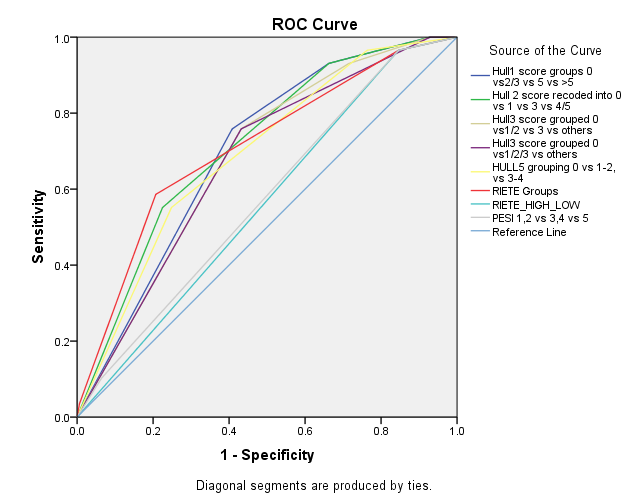 | \| Test Result Variable(s) \| AUC \| P (asympt.) \| \| --- \| --- \| --- \| \| \| Hull1 score groups 0 vs2/3 vs 5 vs >5 \| .698 \| .001 \| \| Hull 2 score recoded into 0 vs 1 vs 3 vs 4/5 \| .715 \| .000 \| \| Hull3 score grouped 0 vs1/2 vs 3 vs others \| .680 \| .002 \| \| Hull3 score grouped 0 vs1/2/3 vs others \| .672 \| .003 \| \| HULL5 grouping 0 vs 1-2, vs 3-4 \| .692 \| .001 \| \| RIETE Groups \| .710 \| .000 \| \| RIETE_HIGH_LOW \| .560 \| .305 \| \| PESI 1,2 vs 3,4 vs 5 \| .572 \| .216 \|   3-month mortality Valid N (listwise)  Positive 29  Negative 169  Missing 36 |
| 6-month mortality |  |
| 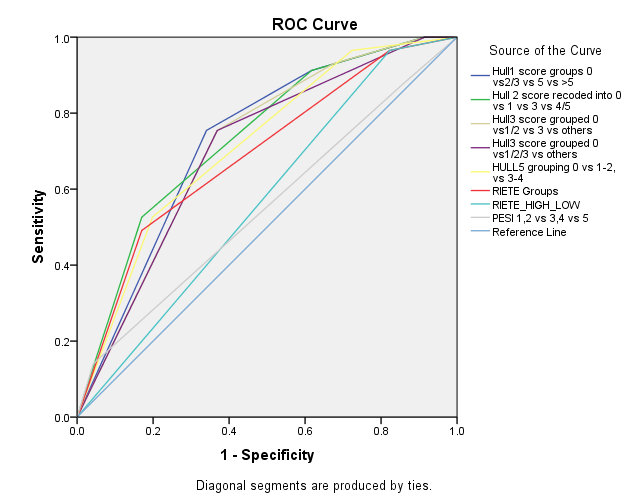 | \| Test Result Variable(s) \| AUC \| P (asympt.) \| \| --- \| --- \| --- \| \| \| Hull1 score groups 0 vs2/3 vs 5 vs >5 \| .729 \| .000 \| \| Hull 2 score recoded into 0 vs 1 vs 3 vs 4/5 \| .736 \| .000 \| \| Hull3 score grouped 0 vs1/2 vs 3 vs others \| .714 \| .000 \| \| Hull3 score grouped 0 vs1/2/3 vs others \| .703 \| .000 \| \| HULL5 grouping 0 vs 1-2, vs 3-4 \| .715 \| .000 \| \| RIETE Groups \| .691 \| .000 \| \| RIETE_HIGH_LOW \| .571 \| .117 \| \| PESI 1,2 vs 3,4 vs 5 \| .551 \| .261 \|   6-month mortality Valid N (listwise)  Positive 57  Negative 141  Missing 36 |

**Table 8**. Candidate Hull Scores with risk groups comparison for 30-day mortality).

| 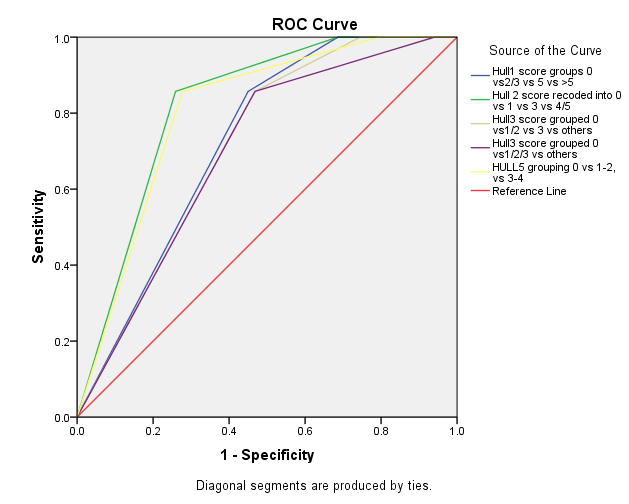 | 30-day Mortality Valid N (listwise)  Positive 7  Negative 220  Missing 7 |
| --- | --- |
| \|  \|  \|  \| Asymptotic 95% CI \| \| \| --- \| --- \| --- \| --- \| --- \| \| Hull1 score groups 0 vs2/3 vs 5 vs >5 \| .726 \| .042 \| (.591, .861) \| \| \| Hull 2 score recoded into 0 vs 1 vs 3 vs 4/5 \| .821 \| .004 \| (.707, .936) \| \| \| Hull3 score grouped 0 vs1/2 vs 3 vs others \| .713 \| .056 \| (.570, .855) \| \| \| Hull3 score grouped 0 vs1/2/3 vs others \| .699 \| .074 \| (.535, .862) \|  \| \| HULL5 grouping 0 vs 1-2, vs 3-4 \| .805 \| .006 \| (.675, .934) \| \| | |

**Table 9.** An attempt to recode the Hull2 score groups into three categories (high-intermediate-low) deprived Hull2 from its statistical significance as shown below. Please note that valid cases list wise are the same as in Table 8.

| Test Result Variable(s) | AUC | P (asympt.) |  |
| --- | --- | --- | --- |
|  |  |  | Asymptotic 95% Confidence Interval |
| HULL5 grouping 0 vs 1-2, vs 3-4 | .805 | .006 | (.675, .934) |
| HULL2 Grouping of 0 vs 1 vs 2/3 | .657 | .158 | (.503, .811) |


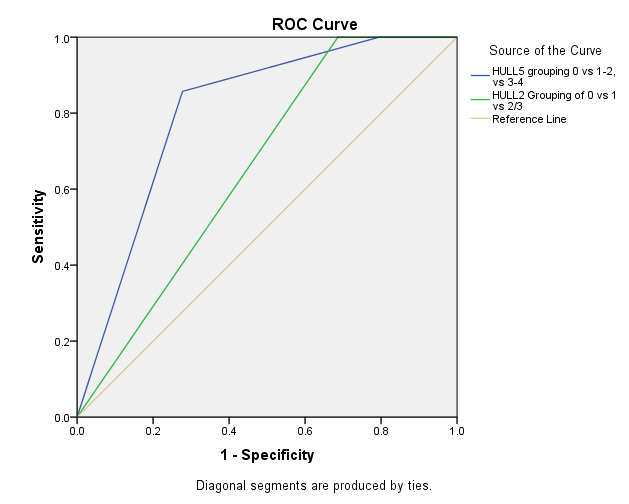


# Appendix D

## **Exploratory analyses per cancer diagnosis.**

[OS: Overal Survival, CI: Confidence Interval, NSCLC: Non-Small Cell Lung Cancer, SCLC: Small Cell Lung Cancer, RCC: Renal Cell Carcinoma]

| **Diagnosis** | **n** | **Median OS (Months)** | **95% CI** |
| --- | --- | --- | --- |
| Pancreaticobiliary | 21 | 4.0 | (1.7, 6.3) |
| Bladder | 8 | 6.0 | (4.7, 7.4) |
| Breast | 21 | 18.7 | (5.2, 32.2) |
| Colorectal | 58 | 19.2 | (11.9, 26.4) |
| Gastric / Oesophageal | 38 | 11.9 | (3.8, 20) |
| Lung (NSCLC/SCLC) | 27 | 5.7 | (5.1, 6.3) |
| Lymphoma | 7 | 8.6 | (7.6, 9.6) |
| Melanoma | 8 | 5.6 | (.0, 24.3) |
| Other | 34 | 14.4 | (8.1, 20.7) |
| RCC | 11 | 21 | NC |

## **Tables 10-14:** Exploratory prognostic factor analyses for survival and Mortality per cancer type were feasible only for the most common diagnoses in our cohort: Colorectal n=58, Gastric/Oesophageal n=38, Lung n=28, Breast=21, Pancreaticobiliary n=21)

[*Log rank **2-sided asymptotic Pearson Chi-Square, OS: Overall Survival, CI: Confidence interval, NC: Non-calculable, NR: Note Reached, PS: Performance status (WHO/ECOG), WCC: White Cell Count, PESI: Pulmonary Embolism Severity Index.]

| **Table 10. Colorectal Cancer** | | | | | | | | | | |
| --- | --- | --- | --- | --- | --- | --- | --- | --- | --- | --- |
|  |  | **Survival** | | **Mortality (events)** | | | | | | |
|  | **n** | **OS (95%CI)** | **P*** | **30-d  n (%)** | **P**** | **3-m  n (%)** | **P**** | **6m  n (%)** | **P**** |  |
| Metastatic/incurable disease |  |  |  |  |  |  |  |  |  |  |
| No Yes | 46 12 | NR 17 (2.6, 11.9) | <.001 | 0 (0) 1 (8) | .606 | 0 (0) 4 (10) | .290 | 1(8) 6 (13) | .656 |  |
| New or Worsening symptoms |  |  |  |  |  |  |  |  |  |  |
| No Yes | 30 28 | 20.1 (NC) 17.7 (9.8, 25.6) | .084 | 0 (0) 1 (8) | .296 | 1 (3) 3 (11) | .268 | 2 (7) 5 (18) | .191 |  |
| WCC |  |  |  |  |  |  |  |  |  |  |
| <11.3 x10^9^/L ≥11.3 x10^9^/L | 54 4 | 19 (14, 24.3) NC | .164 | 1 (2) 0 (0) | .784 | 4 (7) 0 (0) | .573 | 7 (13) 0 (0) | .443 |  |
| Creatinine |  |  |  |  |  |  |  |  |  |  |
| <55μmol/L >55μmol/L | 7 51 | 12.6 (.3, 24.9) 19.2(12, 26.4) | .166 | 1 (14) 0 (0) | .006 | 2 (29) 2 (4) | .016 | 2 (29) 5 (10) | .153 |  |
| PS |  |  |  |  |  |  |  |  |  |  |
| 0 1,2 3,4 | 29 23 4 | 24 (4.2, 44.5) 17.7 (7.6, 27.8)  3.9 | .052 | 1 (3) 0 (0) 0 (0) | .623 | 2 (7) 1 (4) 1 (25) | .333 | 2 (7) 3 (13) 2 (50) | .050 |  |
| PESI |  |  |  |  |  |  |  |  |  |  |
| I,II III,IV V | 4 49 5 | 20.9 (NC) 21 (8.3, 33,6) 5.5 (.0, 11.3) | .006 | 0 (0) 1 (2) 0 (0) | .911 | 0 (0) 2 (4) 2 (40) | .009 | 0 (0) 4 (8) 3 (60) | .002 |  |

| **Table 11. Gastric / Oesophageal Cancer** | | | | | | | | | | |
| --- | --- | --- | --- | --- | --- | --- | --- | --- | --- | --- |
|  |  | **Survival** | | **Mortality (events)** | | | | | | |
|  | **n** | **OS (95%CI)** | **P*** | **30-d  n (%)** | **P**** | **3-m  n (%)** | **P**** | **6m  n (%)** | **P**** |  |
| Metastatic/incurable disease |  |  |  |  |  |  |  |  |  |  |
| No Yes | 15 23 | 24.1 (4.7, 8.5) 6.6 (4.7, 8.5) | .003 | 0 (0) 0 (0) | NC | 1 (7) 4 (17) | .339 | 1 (7) 11 (48) | .008 |  |
| New or Worsening symptoms |  |  |  |  |  |  |  |  |  |  |
| No Yes | 16 22 | 19.4 (11.3, 27.5) 6.6 (4.6, 8.6) | .361 | 0 (0) 0 (0) | NC | 2 (13) 3 (14) | .919 | 3 (19) 9 (41) | .147 |  |
| WCC |  |  |  |  |  |  |  |  |  |  |
| <11.3 x10^9^/L ≥11.3 x10^9^/L | 37 1 | 13.6 (5.6, 21.7) NC | .520 | 0 (0) 0 (0) | NC | 5 (14) 0 (0) | .693 | 12 (32) 0 (0) | .491 |  |
| Creatinine |  |  |  |  |  |  |  |  |  |  |
| <55μmol/L >55μmol/L | 2 36 | 3 (NC) 13.6 (2.6, 24.7) | .073 | 0 (0) 0 (0) | NC | 0 (0) 5 (14) | .572 | 1 (50) 11 (31) | .565 |  |
| ECOG/WHO PS |  |  |  |  |  |  |  |  |  |  |
| 0 1,2 3,4 | 21 14 3 | 23.7 (17.1, 30.3) 7 (2.1, 11.9) 5.5 (3.8, 20) | .012 | 0 (0) 0 (0) 0 (0) | NC | 2 (10) 3 (21) 0 (0) | .464 | 5 (24) 5 (36) 2 (67) | .300 |  |
| PESI |  |  |  |  |  |  |  |  |  |  |
| I,II III,IV V | 3 32 3 | 5.5 (1.6, 9.5) 13.6 (6.1, 21.2) 3.5 (1.9, 5) | .488 | 0 (0) 0 (0) 0 (0) | NC | 0 (0) 4 (13) 1 (33) | .464 | 2 (67) 8 (25) 2 (67) | .131 |  |

| **Table 12. Lung Cancer** | | | | | | | | | | |
| --- | --- | --- | --- | --- | --- | --- | --- | --- | --- | --- |
|  |  | **Survival** | | **Mortality (events)** | | | | | | |
|  | **n** | **OS (95%CI)** | **P*** | **30-d  n (%)** | **P**** | **3-m  n (%)** | **P**** | **6m  n (%)** | **P**** |  |
| Metastatic/incurable disease |  |  |  |  |  |  |  |  |  |  |
| No Yes | 0 27 | NC 5.7 (5.1, 6.3) | NC | 0 3 (11) | NC | 0 8 (29) | NC | 0  15 (54) | NC |  |
| New or Worsening symptoms |  |  |  |  |  |  |  |  |  |  |
| No Yes | 15 12 | 7 (3.8, 10.2) 4.4 (0, 9.1) | .061 | 1 (6) 2 (17) | .378 | 3 (19) 5 (42) | .184 | 7 (44) 8 (67) | .229 |  |
| WCC |  |  |  |  |  |  |  |  |  |  |
| <11.3 x10^9^/L ≥11.3 x10^9^/L | 23 4 | 5.7 (4.5, 7) 2.8 (0, 7.3) | .429 | 2 (8) 1 (25) | .318 | 6 (25) 2 (50) | .306 | 12 (50) 3 (75) | .353 |  |
| Creatinine |  |  |  |  |  |  |  |  |  |  |
| <55μmol/L >55μmol/L | 4 22 | 2.8 (0, 5.9) 5.7 (4.5, 7) | .559 | 0 (0) 3 (13) | .444 | 2 (50) 6 (26) | .334 | 3 (75) 11 (48) | .315 |  |
| PS |  |  |  |  |  |  |  |  |  |  |
| 0 1,2 3,4 | 7 15 4 | 6.5 (4.5, 8.5) 5.6 (2.3, 9) 2.2 (.5, 3.9) | .539 | 0 (0) 2 (13) 1 (25) | .395 | 1 (13) 4 (27) 3 (75) | .077 | 3 (38) 8 (53) 3 (75) | .465 |  |
| PESI |  |  |  |  |  |  |  |  |  |  |
| I,II III,IV V | 3 18 6 | 9.3 (3.4, 15.1) 10.4 (.5, 8.3) 5.7 (5.1, 6.3) | .921 | 0 (0) 3 (17) 0 (0) | .393 | 0 (0) 8 (44) 0 (0) | .045 | 1 (25) 11 (61) 3 (50) | .416 |  |

| **Table 13. Pancreaticobiliary Cancer** | | | | | | | | | | |
| --- | --- | --- | --- | --- | --- | --- | --- | --- | --- | --- |
|  |  | **Survival** | | **Mortality (events)** | | | | | | |
|  | **n** | **OS (95%CI)** | **P*** | **30-d  n (%)** | **P**** | **3-m  n (%)** | **P**** | **6m  n (%)** | **P**** |  |
| Metastatic/incurable disease |  |  |  |  |  |  |  |  |  |  |
| No Yes | 0 21 | NC 4 (1.7, 6.4) | NC | 2 (10) | NC | 7 (33) | NC | 13 (62) | NC |  |
| New or Worsening symptoms |  |  |  |  |  |  |  |  |  |  |
| No Yes | 7 13 | 6.3 (3.2, 9.5) 3.8 (2.7, 4.9) | .291 | 0 (0) 2 (15) | .274 | 2 (29) 4 (31) | .919 | 3 (43) 9 (69) | .251 |  |
| WCC |  |  |  |  |  |  |  |  |  |  |
| <11.3 x10^9^/L ≥11.3 x10^9^/L | 11 8 | 6.3 (2.6, 10.1) 3.8 (1, 6.6) | .177 | 0 (0) 2 (25) | .080 | 3 (27) 3 (38) | .636 | 5 (45) 6 (75) | .198 |  |
| Creatinine |  |  |  |  |  |  |  |  |  |  |
| <55μmol/L >55μmol/L | 4 15 | 2.8 (0, 5.8) 5.2 (1.9, 8.4) | .627 | 0 (0) 2 (11) | .440 | 2 (50) 4 (27) | .372 | 3 (75)  8 (53) | .435 |  |
| ECOG/WHO PS |  |  |  |  |  |  |  |  |  |  |
| 0 1,2 3,4 | 3 16 2 | 26.3 (NC) 3.8 (2, 5.6) .5 (NC) | .010 | 0 (0) 1 (6)  1 (50) | .115 | 0 (0)  6 (38) 1 (50) | .392 | 0 (0) 11 (69) 2 (100) | .040 |  |
| PESI |  |  |  |  |  |  |  |  |  |  |
| I,II III,IV V | 3 17 1 | 11.8 (1.4, 22.2) 3.8 (2.5, 5) 2 (NC) | .051 | 0 (0) 2 (12) 0 (0) | .771 | 0 (0) 6 (35) 1 (100) | .171 | 1 (33) 11 (65) 1 (100) | .425 |  |

| **Table 14. Breast** | | | | | | | | | | |
| --- | --- | --- | --- | --- | --- | --- | --- | --- | --- | --- |
|  |  | **Survival** | | **Mortality (events)** | | | | | | |
|  | **n** | **OS (95%CI)** | **P*** | **30-d  n (%)** | **P**** | **3-m  n (%)** | **P**** | **6m  n (%)** | **P**** |  |
| Metastatic/incurable disease |  |  |  |  |  |  |  |  |  |  |
| No Yes | 0 1 | NC 18.8 (5.2, 32.2) | NC | 0 (0) 0 (0) | NC | 0 (0) 2 (10) | NC | 0 (0) 5 (24) | NC |  |
| New or Worsening symptoms |  |  |  |  |  |  |  |  |  |  |
| No Yes | 9 12 | 26.4 (19.8, 33) 15.4 (28) | .521 | 0 (0) 0 (0) | NC | 1 (11) 1 (8) | .830 | 2 (22) 3 (25)` | .882 |  |
| WCC |  |  |  |  |  |  |  |  |  |  |
| <11.3 x10^9^/L ≥11.3 x10^9^/L | 18 3 | 26.4 (14.2, 38.7)  6.4 (2.1, 10.7) | .027 | 0 (0) 0 (0) | NC | 2 (11) 0 (0) | .544 | 4 (22) 1 (33) | .676 |  |
| Creatinine |  |  |  |  |  |  |  |  |  |  |
| <55μmol/L >55μmol/L | 6 15 | 40 (NC) 18.7 (8.9, 28.6) | .080 | 0 (0) 0 (0) | NC | 0 (0) 2 (13) | .347 | 2 (33) 3 (20) | .517 |  |
| ECOG/WHO PS |  |  |  |  |  |  |  |  |  |  |
| 0 1,2 3,4 | 10 8 3 | 23.2 (12.6, 33.9) 40.5 (NC) 2.9 (1.8, 4) | .042 | 0 (0) 0 (0) 0 (0) | NC | 0 (0) 0 (0) 2 (67) | .001 | 0 (0) 2 (25) 3 (100) | .002 |  |
| PESI |  |  |  |  |  |  |  |  |  |  |
| I,II III,IV V | 3 17 1 | NC NC NC | .024 | 0 (0) 0 (0) 0 (0) | NC | 0 (0) 2 (12) 0 (0) | .771 | 0 (0) 5 (29) 0 (0) | .462 |  |
